# Independent Association Between Frailty and Fear of Falling in Older Adults After Hip Fracture Surgery: A Cross-Sectional Study Using a Secondary Analysis

**DOI:** 10.1155/nrp/2175740

**Published:** 2025-01-03

**Authors:** YoungJi Ko, JungAh Park, Seung-Hoon Baek

**Affiliations:** ^1^Department of Nursing, Daegu Haany University, Gyeongsan-Si, Gyeongsangbuk-Do, Republic of Korea; ^2^Department of Nursing, CHA University, Seoul, Republic of Korea; ^3^Department of Orthopedic Surgery, Kyungpook National University, Kyungpook National University Hospital, Daegu, Republic of Korea

**Keywords:** fear, frail elderly, frailty, hip fractures

## Abstract

**Purpose:** This study aimed to investigate the independent association between the fear of falling (FOF) and frailty in Korean older adults after hip fracture surgery.

**Methods:** The study included 149 participants. Participants were asked to provide general characteristics and complete walking speed, grip strength, frailty, and Short Falls Efficacy Scale-International assessments. A binary logistic regression model was used to investigate the independent association of the FOF with frailty.

**Results:** Among the participants, 49% were found to be prefrail and 24.8% were frail. Additionally, 86.5% reported a moderate to severe FOF. Participants with a FOF were 1.23 times more likely to be frail, and those aged 85 years and older were 13.25 times more likely to be frail.

**Conclusion:** This study's findings serve as a basis for developing and applying interventions to reduce frailty in older adults after hip fracture surgery. These interventions should consider the oldest-old (≥ 85 years) age group when they are designed.

## 1. Introduction and Objective

Hip fractures, particularly in the aging population, are a critical healthcare concern globally. Projections indicate that the annual incidence of hip fractures will rise from approximately 0.98 million in 1990 to an estimated 1.87 million by 2050 [1]. Notably, most hip fractures occur in females, predominantly affecting those aged 80 years and older [1–3]. While hip fracture surgery is the preferred treatment, the 1-year mortality rate after surgery is high at about 22.4% globally [1], with a similar trend observed in South Korea, where the rates are 21% for men and 15% for women [4].

Previous studies [2, 5, 6] have indicated that older adults with frailty experience significantly higher mortality rates, longer hospital stays, and increased total hospital costs after hip fracture surgery compared with those without frailty. Frailty, which is frequently observed in older adults with hip fractures, is intricately linked with poor bone health and an increased risk of falls. This connection supports the existence of a robust causal relationship between frailty, falls, fractures, and adverse postoperative outcomes, highlighting the critical need for comprehensive management strategies in this vulnerable population [3, 6].

Fried et al. [7] defined a phenotype of frailty to include four or more of the following criteria: unintentional weight loss, exhaustion, weakness, slow walking speed, and low physical activity. Some studies have shown that up to 91% of older adults with hip fractures exhibit either frailty or prefrailty [5, 8]. Given the high mortality rate associated with these conditions, early detection of frailty in older adults with hip fractures is essential because of its significance in predicting severe health consequences. Factors, such as advanced age, multiple underlying diseases, and low body mass index, are known risks for frailty and prefrailty in older adults with hip fractures [9].

Recent studies [10–14] have highlighted the significant association between psychological factors and physical frailty. Higher levels of depression have been associated with more severe frailty [10, 12]. Moreover, Kim et al. [11] found that depression notably affected the association between frailty and the risk of falls. In addition, depression has been linked to exhaustion as a component of frailty [7, 10, 11]. This evidence underscores the need for nursing care to address the physical and psychological aspects of frailty.

Moreover, the fear of falling (FOF) is a prevalent psychological challenge among older adults after hip fracture surgery, occurring in approximately 49% at 4–6 months [15] and 47.5% at 6–12 months [12] after surgery. The FOF leads to fear-related activity avoidance [12, 14, 16] and decreases balance confidence and physical performance, increasing the risk of recurrent falls [17].

Recent longitudinal studies have demonstrated a bidirectional association between the FOF and frailty among community-dwelling older adults. For example, Mo et al. [14] found that the FOF predicted prefrailty and frailty. In a 48-month cohort study, Makino et al. [13] found that community-dwelling older adults who were prefrail or frail had a significantly higher risk of developing FOF. These findings suggest that interventions aimed at reducing the FOF, including exercise programs, may mitigate frailty [14, 16]. The FOF following hip fractures may exacerbate pre-existing frailty [3, 6], and interventions that reduce the FOF may have the potential to reduce frailty severity in later life.

To our knowledge, the association between FOF and frailty in Korean adults 65 years and older after hip fracture surgery has not yet been studied. Therefore, this study aimed to describe and investigate the independent association between FOF and frailty in Korean older adults after hip fracture surgery.

## 2. Methods

### 2.1. Research Design and Ethical Considerations

This study was a secondary analysis of data from a research project (No: 2022-01-022-001), which aimed to investigate the prevalence of FOF and associated factors. This study comprised a retrospective data analysis to explore the frailty, walking speed, grip strength, and the FOF among older adults after undergoing hip fracture surgery. The Institutional Review Board of K hospital (No: 2024-04-024) approved this study, and all participants provided written informed consent to participate. Confidentiality and privacy were guaranteed, and each data file was coded and anonymized.

### 2.2. Participants and Data Collection

Data were collected between April 2022 and July 2023 by two trained interviewers. The data were initially collected from older adults who visited the orthopedic outpatient clinic of a teaching hospital and agreed to participate in the primary study (manuscript in preparation). This study used the sample characteristics, frailty, and FOF data collected from the same participants. Inclusion criteria for the older adults included (1) over 65 years old, (2) 1–5 years after hip fracture surgery, (3) an understanding of the study's objectives, and (3) the willingness to provide informed consent. The exclusion criteria included older adults who were (1) incapable of following instructions because of severe neurological disorders, such as a stroke or dementia, (2) unable to walk despite using walking aids, (3) hospitalized, (4) taking psychiatric drugs, and (5) required or underwent revision surgery for hip fractures.

## 3. Measures

### 3.1. Sample Characteristics

The participants' general characteristic data included gender, age, falling history, level of pain, and type of surgery. The question, “How many times have you fallen in the past year?” was used to assess the history of falls. Pain levels were evaluated with a single question, “Are you currently in pain?” with a 5-point Likert scale. The information regarding age and type of surgery was collected from medical records.

Walking speed was gauged using a 6-meter usual walk test as described by Chen et al. [18]. Participants were asked to begin at a taped line with their toes aligned with the edge, walk at their normal pace, and continue several steps past the taped finish line [19]. Trained personnel recorded the time to complete the course from the start to the stop line using a stopwatch. The walking assessment took place in a designated area of the orthopedic clinic. Total course time was subsequently converted to meters per second (m/s). A walking speed of 0.8 m/s or less was considered indicative of low physical performance [18].

Grip strength was measured using a dynamometer (TKK5401, Takei Scientific Instruments Co., Ltd., Niigata, Japan). A trained research assistant asked participants to sit in a chair with their arms not resting on the chair handle and encouraged them to flex their elbow to 90° when gripping the dynamometer so that the second joint of the fingers was at a right angle and then to exert maximum force for 4-5 s. The higher of two measurements taken in the same manner after a 60 s rest period was used [18, 20]. Grip strength of 26 kg or less in men and 18 kg or less in women is considered indicative of low handgrip strength [18].

### 3.2. Frailty

The fatigue, resistance, ambulation, illnesses, and loss of weight (FRAIL) instrument, originally developed by Jung et al. [21] and translated into Korean (K-FRAIL) and validated by Morley, Malmstrom, and Miller [22], was assessed to evaluate frailty. A higher score indicates more severe frailty, with scores categorized as follows: 0 as robust, 1-2 as prefrail, and 3–5 as frail; these scores are significantly associated with the phenotype of frailty proposed by Fried et al. [7] and differentiate vulnerability from robustness with high sensitivity (0.90) and lower specificity (0.33) [22].

### 3.3. FOF

The Korean version [23] of the Short Falls Efficacy Scale-International (FES-I), originally developed by Kempen et al. [24], was used to evaluate the FOF. This scale includes seven items with a 4-point Likert scale, ranging from 7 (no concern about falling) to 28 (severe concern about falling). Based on the scores, three levels of concern are categorized, namely, low (7-8 points), moderate (9–13 points), and high (14–28 points). The reliability and validity of the scale were confirmed by Ko, Lee, and Baek [23] and Cronbach's *α* coefficient for this study was 0.93.

### 3.4. Data Analysis

The SPSS Statistics software (v.23.0, IBM Statistical Software, IBM Corp. Armonk, NY, USA) was used to analyze the data. In the descriptive analyses for continuous variables, the mean (M) and standard deviation (SD) were presented; frequencies and percentages were presented for categorical variables. Differences in sample characteristics between participants with or without frailty were determined using an independent *t*-test for continuous variables, with the resulting *t*-value, and the chi-squared test for categorical variables, with the *Z*-value. In addition, a binary logistic regression model was used to investigate the independent association of the FOF with frailty. Scores of 0–2, representing robust and prefrail participants, were categorized as nonfrailty, while scores of 3-5, representing frail participants, were categorized as frailty in the analysis. Significant variables associated with frailty were included in the regression model. The statistical significance level was set at *p* < 0.05 for all analyses.

## 4. Results

The 149 older adults in this study had a mean age of 78.03 years (SD = 6.81). Approximately 73.8% of the participants were female. Of the 149 participants, 22.2% reported falling twice or more in the past year and 62.4% had undergone internal fixation after a hip fracture. The average 6-meter gait speed for all participants was 0.65 ± 0.33 m/s and 101 participants (67.8%) had a gait speed of ≤ 0.8 m/s, which is indicative of impaired physical performance. The average grip strength for all participants was 24.07 kg (SD = 13.28 kg); 84 participants (56.4%) had low handgrip strength, defined as < 26 kg for men and < 18 kg for women (Table 1). The mean FOF score for all participants was 15.81 ± 5.72, with 96 participants (64.4%) classified as having a high FOF concern according to their Short FES-I score. The mean frailty index was 1.63 ± 1.24 for all participants, with 49% of participants classified as prefrail and 24.8% as frail (Table 2).

Among the participants, age, falling history, pain, 6-meter gait speed, and grip strength were significantly associated with frailty. However, there was no association between frailty and the other characteristics, including sex and type of hip surgery (Table 3). The variables significantly associated with frailty were entered into a logistic regression model to identify the association between the FOF and frailty. After adjusting for age, falling history, pain, 6-meter gait speed, and grip strength, older adults with a FOF were 1.23 times more likely to be frail (odds ratio (OR) = 1.23, 95% confidence interval (CI) = 1.10–1.39; *p* < 0.001). In addition, participants aged 85 and older (OR = 13.25, 95% CI = 1.73–101.38; *p*=0.013) and those with low handgrip strength (OR = 1.04, 95% CI = 1.00–1.08; *p*=0.018) were more likely to be frail (Table 4).

## 5. Discussion

This study found that 24.8% of the participants exhibited frailty, with a mean K-FRAIL scale index of 1.63 (SD = 1.24) [21]; this is in close agreement with the findings of Lee et al. [25], who reported that 23% of participants in a Korean rural community setting were found to be frail. However, this study's findings are significantly higher than the frailty rate (17.5%) and mean frailty index (0.19 ± 0.17), determined using the same tool, observed in Korean older adults who visited an outpatient clinic and ward [21]. In contrast, a study by Mangione et al. [8] found that 59% of participants were prefrail and 32% were frail after hip fracture surgery, which is higher than this study's findings and those of Lee et al. [25]. These variations can be attributed to the timing of the assessments after hip fracture surgery (3 months vs. 1–5 years) and the use of different evaluation tools (Fried et al.‘s [7] criteria vs. the K-FRAIL scale [21, 22]). This study's findings suggest that frailty is highly prevalent in those who have undergone hip fracture surgery and should be identified before discharge. Frailty serves as a predictive factor for community ambulation and mortality [2, 6, 8], suggesting that recognizing frailty should influence nursing plans.

Our participants were able to walk with or without walking devices, and 86.5% of these participants experienced more than moderate concern about falls. Therefore, the FOF results were much higher than those reported in previous studies [12, 26] for independent community-dwelling older adults [12, 16, 17, 26], most likely because the majority of participants experienced hip fractures due to simple falls, which can cause FOF. Additionally, in this study, the participants' advanced age [1] and female gender [1, 12, 16] in the phenotype of hip fracture are likely to have increased the FOF.

Furthermore, our findings indicate that the FOF is associated with a 1.23 times increased risk of frailty in older adults after hip fracture surgery, even after adjusting for age [2, 9, 14, 27], gender [14], falling history [14, 27], pain [14], gait speed [13], and grip strength [13], which can potentially contribute to frailty. A recent systematic review [28] found that adjusted ORs for the FOF increased the risk of frailty from 1.04 to 7.16. In a study of independent older adults living in the community, the FOF was linked to a 7.2-fold higher risk of becoming frail [27]. These wide variations are likely due to differences in the tools for assessing frailty and the FOF [6, 28], participants' characteristics, and statistical approaches for data analysis [28]. Regardless of these variations, the results of this study clarify the association of FOF with frailty and suggest that FOF exacerbates frailty in older adults after hip fracture surgery.

In addition to the FOF, age was an important, nonmodifying factor of frailty, with the oldest-old (≥ 85 years) participants being associated with a 13.25 times increased risk of frailty. Low grip strength, which has been evaluated as a measure of frailty [13], was associated with a 1.04 times increased risk of frailty. However, this study's results did not confirm that the criteria of sarcopenia in Asia [18], particularly the criterion of low handgrip strength, significantly affected frailty. Therefore, further studies with larger sample sizes are needed to validate the low grip strength values (26 kg or less in men and 18 kg or less in women) in the criteria of sarcopenia in Asia [18]. Moreover, the 6-meter gait speed, another measure for evaluating physical performance [13], did not significantly affect frailty after including the FOF in the final data analysis. Possible reasons are that the Short FES-I [24] already includes physical performance and the FOF can lead to functional decline [28], potentially decreasing physical performance's influence on frailty compared with the FOF.

Both FOF and frailty are critical factors leading from self-care to disability [6, 8, 28] after hip fracture surgery. It is crucial to evaluate frailty and also identify the FOF in older adults after hip fracture surgery. However, the FOF is not included in comprehensive geriatric assessments, which are challenging to perform regularly because of South Korea's limited resources [21]. Therefore, easy, quick, and reliable screening tools, including the Short FES-I and K-FRAIL, are necessary to identify frailty and FOF in acute care settings.

This study has some limitations. First, the cross-sectional design cannot explain the causal association between the FOF and frailty. Second, the relatively small sample size based on the primary study could not determine whether the cutoff scores of the Short FES-I [24] and grip strength [18] assessments were associated with frailty. Therefore, studies with larger sample sizes and longitudinal designs are needed to verify this study's results. Third, despite controlling for several confounding factors, others such as social aspects were not considered. However, to the best of our knowledge, this study is one of the first to investigate the association between FOF and frailty, adjusting for potential confounding factors, in older adults after hip fracture surgery. This study's findings indicate a significant relationship between FOF and frailty, suggesting that FOF may be an important factor to consider in understanding frailty. They highlight that interventions for reducing the FOF are essential for older adults after hip fracture surgery.

## 6. Conclusion

The FOF and frailty are major concerns in older adults with hip fractures. This study identified the association between the FOF and frailty in older adults after hip fracture surgery and demonstrated that the FOF can exacerbate frailty. These findings provide a basis for developing and implementing interventions including tailored exercise programs to reduce frailty and FOF in older adults after hip fracture surgery. Additionally, the oldest-old age group should be taken into consideration when planning these interventions.

## Figures and Tables

**Table 1:** Sample characteristics.

Characteristics	Category	Number (%) of patients (*N* = 149) M ± SD
Sex	Male	39 (26.2)
	Female	110 (73.8)

Age (years)	65–74	46 (30.9)
	75–84	79 (53.0)
	≥ 85	24 (16.1)
	Average	78.03 ± 6.81

Falling history (*n*)	0	49 (32.9)
	1	67 (45)
	2	19 (12.8)
	≥ 3	14 (9.4)

Pain	Not at all	64 (43)
	A little bit	56 (37.6)
	Moderate	24 (16.1)
	Severe	5 (3.3)

Type of hip surgery	Internal fixation	93 (62.4)
	Arthroplasty	56 (37.6)

6-meter gait speed	Average	0.65 ± 0.33 m/s
	≤ 0.8 m/s	101 (67.8)
	> 0.8 m/s	48 (32.2)

Grip strength	Average	24.07 ± 13.28 kg
	< 26 kg in men, 18 kg in women	84 (56.4)
	≥ 26 kg in men, 18 kg in women	65 (43.6)

*Abbreviation:* M ± SD, mean ± standard deviation.

**Table 2:** Results from the fear of falling (FOF) and frailty.

Variable	Category	Number of patients (%) (*N* = 149)	Score (M ± SD)	Range
FOF (Short FES-I)			15.81 ± 5.72	7–28
	Low concern (7–8)	20 (13.5)		
	Moderate concern (9–13)	33 (22.1)		
	High concern (14–28)	96 (64.4)		

Frailty (K-FRAIL)			1.63 ± 1.24	0–5
	Robust (0)	39 (26.2)		
	Prefrailty (1–2)	73 (49.0)		
	Frailty (3–5)	37 (24.8)		

*Abbreviations:* K-FRAIL, Korean fatigue, resistance, ambulation, illnesses, and loss of weight scale; M ± SD, mean ± standard deviation; Short FES-I, Short Falls Efficacy Scale-International.

**Table 3:** Relationships between frailty and sample characteristics.

Variable	Category	Frailty (*N* = 149)	
		*t*§ or *Z*†	*p*
Sex^†^	Male		0.052^a^
	Female		
Age^†^	65–74	10.54	0.005⁣^∗∗^
	75–84		
	≥ 85		

Falling history (*n*)^†^	0	10.26	0.016⁣^∗^
	1		
	2		
	≥ 3		

Pain^†^	Not at all	23.60	< 0.001⁣^∗∗∗^
	A little bit		
	Moderate		
	Severe		

Type of hip surgery^†^	Internal fixation		0.328^a^
	Arthroplasty		

6-meter gait speed^†^	Average	4.53^§^	< 0.001⁣^∗∗∗^
	≤ 0.8 m/s		0.001⁣^∗∗^^a^
	> 0.8 m/s		
Grip strength^†^	Average	−2.36^§^	0.022⁣^∗^
	< 26 kg in men, 18 kg in women		0.129^a^
	≥ 26 kg in men, 18 kg in women		

*Abbreviation:* M ± SD, mean ± standard deviation.

^†^Chi-square test.

^§^ *t*-tests.

^a^Fisher's exact test.

⁣^∗^*p* < 0.05.

⁣^∗∗^*p* < 0.01.

⁣^∗∗∗^*p* < 0.001.

**Table 4:** Binary logistic regression model for frailty.

Variable (*N* = 149)	*β*	*p*	Crude odds ratio (OR) (95% CI)
Age			
65–74^a^			
75–84	1.45	0.185	4.26 (0.50–36.30)
≥ 85	2.58	0.013⁣^∗^	13.25 (1.73–101.38)
Falling history			
0^a^			
1	−0.38	0.745	0.68 (0.67–6.94)
2	0.25	0.814	1.28 (0.15–10.54)
≥ 3	1.76	0.135	5.86 (0.57–59.51)
Pain			
Not at all^a^			
A little bit	−21.42	0.999	0.00
Moderate	−20.05	0.999	0.00
Severe	−20.28	0.999	0.00
6-meter gait speed			
≤ 0.8 m/s	−1.06	0.140	0.34 (0.08–1.41)
> 0.8 m/s^a^			
Grip strength (average)	0.04	0.018⁣^∗^	1.04 (1.00–1.08)
Fear of falling (FOF, average)	0.21	< 0.001⁣^∗∗∗^	1.23 (1.10–1.39)

^a^The reference group.

⁣^∗^*p* < 0.05.

⁣^∗∗^*p* < 0.01.

⁣^∗∗∗^*p* < 0.001.

## Data Availability

The data that support the findings of this study are available upon request.

## References

[1] Sing C., Lin T., Bartholomew S. (2023). Global Epidemiology of Hip Fractures: Secular Trends in Incidence Rate, Post‐Fracture Treatment, and All‐Cause Mortality. *Journal of Bone and Mineral Research*.

[2] Gandossi C. M., Zambon A., Ferrara M. C (2023). Frailty and Post-Operative Delirium Influence on Functional Status in Patients With Hip Fracture: The GIOG 2.0 Study. *Aging Clinical and Experimental Research*.

[3] Martin F. C., Ranhoff A. H. (2021). Frailty and Sarcopenia. *Practical Issues in Geriatrics*.

[4] Kim B., Lim J., Ha Y. (2020). Recent Epidemiology of Hip Fractures in South Korea. *Hip Pelvis*.

[5] Kwak M. J., Digbeu B. D., Des Bordes J., Rianon N. (2022). The Association of Frailty With Clinical and Economic Outcomes Among Hospitalized Older Adults With Hip Fracture Surgery. *Osteoporosis International*.

[6] Song Y., Wu Z., Huo H., Zhao P. (2022). The Impact of Frailty on Adverse Outcomes in Geriatric Hip Fracture Patients: A Systematic Review and Meta-Analysis. *Frontiers in Public Health*.

[7] Fried L. P., Tangen C. M., Walston J. (2001). Frailty in Older Adults: Evidence for a Phenotype. *The Journals of Gerontology Series A: Biological Sciences and Medical Sciences*.

[8] Mangione K. K., Craik R. L., Kenny A. (2021). The Effect of Frailty on Walking Recovery after Hip Fracture: A Secondary Analysis of the Community Ambulation Project. *The Journals of Gerontology: Series A*.

[9] Li Y., Liu F., Xie H., Zhu Y. (2023). Investigation and Analysis of Frailty and Nutrition Status in Older Adult Patients With Hip Fracture. *Nutrition in Clinical Practice*.

[10] Borges M. K., Aprahamian I., Romanini C. V. (2021). Depression as a Determinant of Frailty in Late Life. *Aging & Mental Health*.

[11] Kim Y., Yao Y., Lee S. (2022). Association of Frailty With Fall Events in Older Adults: A 12-Year Longitudinal Study in Korea. *Archives of Gerontology and Geriatrics*.

[12] Lee S., Baek S., Ko Y. (2023). A Comparison of Fear of Falling in Older Adults With Physical Independence and Those With Hip Fracture Surgery. *The Korean Journal of Rehabilitation Nursing*.

[13] Makino K., Lee S., Bae S. (2021). Prospective Associations of Physical Frailty With Future Falls and Fear of Falling: A 48-Month Cohort Study. *Physical Therapy*.

[14] Mo C., Peng W., Luo Y., Tang S., Liu M. (2023). Bidirectional Relationship Between Fear of Falling and Frailty Among Community-Dwelling Older Adults: A Longitudinal Study. *Geriatric Nursing*.

[15] Jaatinen R., Luukkaala T., Hongisto M. T., Kujala M. A., Nuotio M. S. (2022). Factors Associated With and 1-Year Outcomes of Fear of Falling in a Geriatric Post-Hip Fracture Assessment. *Aging Clinical and Experimental Research*.

[16] Merchant R. A., Chen M. Z., Wong B. L. L. (2020). Relationship Between Fear of Falling, Fear‐Related Activity Restriction, Frailty, and Sarcopenia. *Journal of the American Geriatrics Society*.

[17] Hadjistavropoulos T., Delbaere K., Fitzgerald T. D. (2011). Reconceptualizing the Role of Fear of Falling and Balance Confidence in Fall Risk. *Journal of Aging and Health*.

[18] Chen L., Liu L., Woo J. (2014). Sarcopenia in Asia: Consensus Report of the Asian Working Group for Sarcopenia. *Journal of the American Medical Directors Association*.

[19] Lyons J. G., Heeren T., Stuver S. O., Fredman L. (2015). Assessing the Agreement between 3-Meter and 6-Meter Walk Tests in 136 Community-Dwelling Older Adults. *Journal of Aging and Health*.

[20] Hofmann M., Schober-Halper B., Oesen S (2016). Effects of Elastic Band Resistance Training and Nutritional Supplementation on Muscle Quality and Circulating Muscle Growth and Degradation Factors of Institutionalized Elderly Women: The Vienna Active Ageing Study (VAAS). *European Journal of Applied Physiology*.

[21] Jung H., Yoo H., Park S. (2016). The Korean Version of the FRAIL Scale: Clinical Feasibility and Validity of Assessing the Frailty Status of Korean Elderly. *Korean Journal of Internal Medicine (English Edition)*.

[22] Morley J. E., Malmstrom T. K., Miller D. K. (2012). A Simple Frailty Questionnaire (FRAIL) Predicts Outcomes in Middle Aged African Americans. *The Journal of Nutrition, Health & Aging*.

[23] Ko Y., Lee S., Baek S. (2022). Korean Version of the Short Falls Efficacy Scale-International (FES-I): Study of Validity and Reliability. *J Korean Gerontol Nurs.*.

[24] Kempen G. I., Yardley L., Van Haastregt J. C. (2007). The Short FES-I: A Shortened Version of the Falls Efficacy Scale-International to Assess Fear of Falling. *Age and Ageing*.

[25] Lee H., Chong J., Jung H., Baek J. Y., Lee E., Jang I. (2021). Association of the FRAIL Scale With Geriatric Syndromes and Health-Related Outcomes in Korean Older Adults. *Annals of Geriatric Medicine and Research*.

[26] Kuo C., Chen D., Chen Y., Chen P. (2021). Validation of the Short Falls Efficacy Scale-International for Taiwanese Community-Dwelling Older Adults: Associations With Fall History, Physical Frailty, and Quality of Life. *Geriatric Nursing*.

[27] Qin Y., Li J., McPhillips M., Lukkahatai N., Yu F., Li K. (2021). Association of Fear of Falling With Frailty in Community‐Dwelling Older Adults: A Cross‐Sectional Study. *Nursing and Health Sciences*.

[28] de Souza L. F., Canever J. B., Moreira BdS., Danielewicz A. L., de Avelar N. C. P. (2022). Association Between Fear of Falling and Frailty in Community-Dwelling Older Adults: A Systematic Review. *Clinical Interventions in Aging*.

